# Temporomandibular Disorders: “Occlusion” Matters!

**DOI:** 10.1155/2018/8746858

**Published:** 2018-05-15

**Authors:** Robert J. A. M. de Kanter, Pasquale G. F. C. M. Battistuzzi, Gert-Jan Truin

**Affiliations:** Department of Dentistry, Radboud University Medical Center, 6500 HB Nijmegen, Netherlands

## Abstract

By analogy with the journal's title Pain Research and Management, this review describes TMD Research and Management. More specific are the (1) research aspects of “occlusion,” still one of the most controversial topics in TMD, and (2) as much as possible evidence-based management aspects of “TMD” for the dental practitioner. *Research*. The disorders temporomandibular dysfunction and the synonymous craniomandibular dysfunction are still being discussed intensely in the literature. Traditionally, attention is mostly devoted to occlusion and its relationship with these disorders. The conclusions reached are often contradictory. Considering the definitions of temporomandibular and craniomandibular dysfunctions/disorders and “occlusion,” a possible explanation for this controversy can be found in the subsequent methodological problems of the studies. Based on a Medline search of these terms over the past 40 years related to contemporary terms such as “Evidence Based Dentistry” and “Pyramid of Evidence,” these methodological aspects are examined, resulting in recommendations for future research and TMD-occlusal therapy. *Management*. To assist the dental practitioner in his/her daily routine to meet the modern standards of best practice, 7 guidelines are formulated that are explained and accompanied with clinical examples for an evidence-based treatment of patients with this disorder in general dental practices.

## 1. Introduction: Research Section

To date, over 22,000 papers are published concerning the disorders temporomandibular dysfunction and the synonymous craniomandibular dysfunction. In this paper, the term “Temporomandibular Disorders,” henceforth “TMD,” is used to present a collection of the 4 studied terms and its abbreviations: temporomandibular disorders, temporomandibular dysfunction (TMD) and craniomandibular disorders, and craniomandibular dysfunction (CMD).

Recently, a paradigm shift regarding “TMD” has occurred from the biomedical model, more specifically from occlusion, to a biopsychosocial model of disease. The biopsychosocial model was introduced in medicine in 1977 and published in 1978 by Engel [1, 2]. The model was based on general systems and intended to provide a total framework in which all the levels of organization pertinent to health and disease could be conceptualized. One of the levels of organization in the musculoskeletal pain condition “TMD” is the entity “occlusion.” This paper addresses “occlusion” because the interaction between occlusion and “TMD” still has not been unambiguously clarified, leading to controversial research conclusions. This review paper aims to clarify the existing controversy with a scientific approach of the literature in order to provide (1) recommendations for future research and (2) up-to-date evidence-based tools for “TMD” management in the general dental practice.

## 2. Materials and Methods

**Figure 1:**
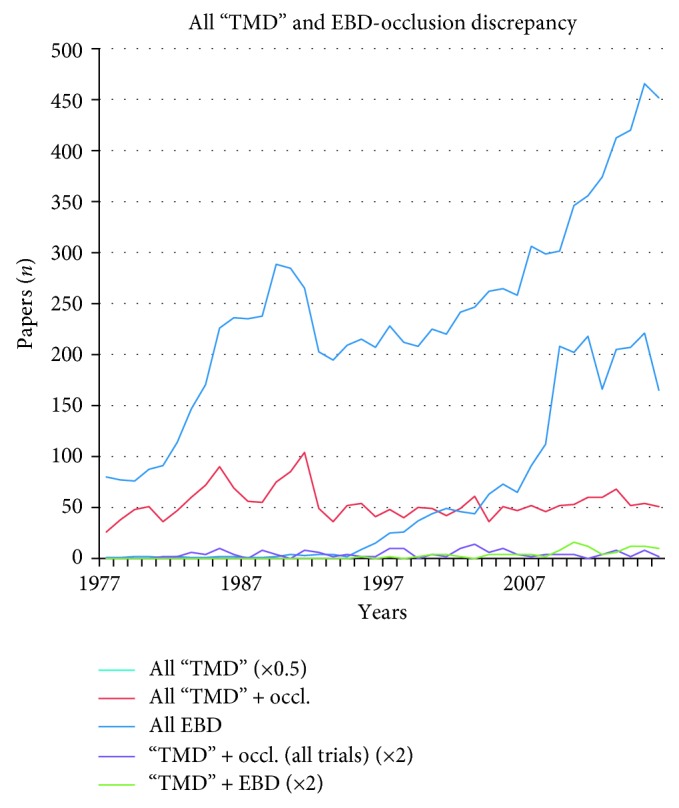
Web of Science search in the Medline database in the period 1977–2016 showing curves of all “TMD” papers (0.5 × all “TMD”), all evidence-based dentistry (EBD) papers, all “ TMD” and “occlusion” papers, the “TMD” + EBD papers, and “TMD” + “occlusion” all trials papers.

**Figure 2:**
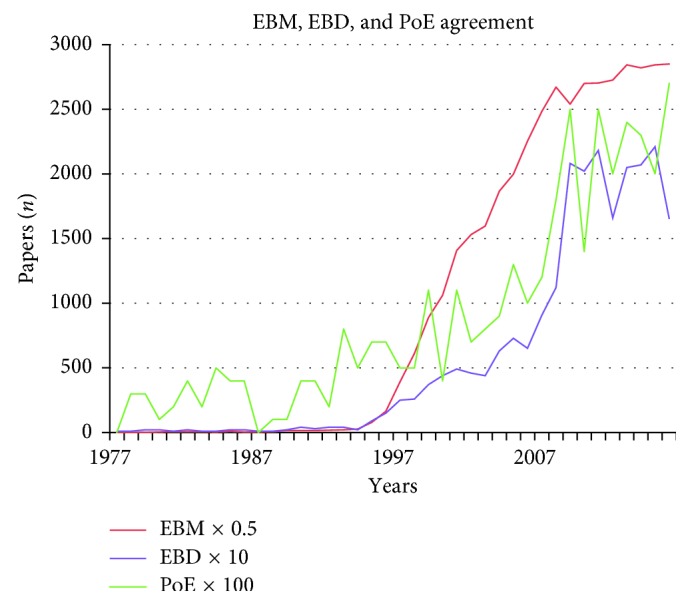
Web of Science search in the Medline database in the period 1977–2016 showing curves of all evidence-based medicine (EBM × 0.5) papers, all evidence-based dentistry (EBD × 10) papers, and all pyramid of evidence (PoE × 100) papers.

In this study, a two-track scientific approach was followed. Literature searches were executed focusing on (1) randomized controlled trials, the highest level in the pyramid of evidence, and all trials and (2) the search terms “evidence based dentistry,” “biopsychosocial model,” and “occlusion.” Web of Science searches in the Medline database were executed over, respectively, a 67-year period for the data in Tables 1 and 3 (1950–2017) and a 40-year period for the data in Table 2 and Figures 1 and 2 (1977–2017). All searches were executed in December 2017.

Search terms and topics were Craniomandibular Disorders, Craniomandibular Dysfunction, Temporomandibular Disorders, Temporomandibular Dysfunction, CMD, TMD, Occlusion, Biopsychosocial Model, Evidence Based Medicine, Evidence Based Dentistry, and Pyramid of Evidence. Results were further filtered by the type of the study (clinical trial, controlled clinical trial, and randomized controlled trial) and/or sorted by the year of publication and frequency of citation. The results of the “occlusion” searches are presented in 3 tables, 2 figures, and an eight-point summary.

## 3. Results

During the recent 67-year period, there are only 35 papers published concerning the biopsychosocial model and “TMD,” 86 different trials, of which 52 randomized controlled trials (Table 2) [3–54] and 21 different studies with the keywords “Evidence Based Dentistry” (Table 3) [55–75] focused on all 6 “TMD” terms and “occlusion.” Further refining the 35 BPSM studies with the search term “evidence based dentistry” results in 3 studies by Ohrbach and Dworkin [76], Simmons [77], and Goldstein [78]. One of the 35 BPSM papers is an RCT by Andrew et al. [79].

It is almost impossible to abstract an extensive RCT into single keywords regarding the results of the study. Nevertheless, the combination of the columns “characteristics of the trial” and “effect/result” is an attempt to realize this. Further explanations of the study results are presented in Discussion. Researchers are invited to further scan, screen, and study the collected papers by themselves to verify the presented conclusions.

Without underestimating the value of papers that will not be addressed in further detail here, the search, presented in Table 3, revealed a number of papers to pay more attention to. First of all, the review papers by Ash [55], both papers by Carlsson [58, 59], the paper of Moreno-Hay and Okeson [69], and the meta-analysis by Fricton et al. [65] are of particular interest. These papers should not only be cited in all future TMD literatures but should also be included in any future study design or at least in Discussion. Of course, the annual reviews of the American Academy of Restorative Dentistry by Donovan et al. [62, 63] are very informative and must have been “a hell of a job” to compose for the experts. Unfortunately, they describe, with all due respect to the 8 authors, only a selection of the available papers. The 2014 review concerning TMD and occlusion refers to 17 papers, and the 2016 review refers to 22 papers including the bruxism section. The total number of TMD papers in the included years of their study is, respectively, 825 (in 2013) and 932 (in 2015). In conclusion, less than 0.5% of all TMD papers are documented and discussed. In the result section of the abstract in 2016, the authors formulate the following: *“The reviews are not meant to stand alone but are intended to inform the interested reader about what has been discovered in the past year. The readers are then invited to go to the source if they wish more detail.”* On one hand, this selection is most probably beneficial for the dental practitioner. On the other hand, a researcher following this advice is directed to only 0.5% of the preselected TMD papers and misses the other 99.5%. Also, the Luther study [68] might have been of interest for our study topic. However, the paper was not specifically focused on RCTs, and its conclusions are based on papers of a lower level of evidence.

Finally, 5 of the selected papers in this search are letters or comments, with 4 of them disputing the Rinchuse 2005 orthodontic-orientated TMD papers. All of the letters and comments concerned about orthodontic-related TMD aspects.

In the period 1977–2017, a total of 20,340 “TMD” papers were published, starting with 160 papers in 1977 to almost a thousand (903) in 2016.  Figure 1 shows a temporary increase in the period 1982–1992. A similar increase seems to be present in the papers about “TMD” refined with “occlusion.” Most “TMD”-“occlusion” papers were published in 1985 (90) and 1991 (104). In that 11-year period, on average, 70 papers were published yearly.

The “TMD” refined with “occlusion” curve does not follow the curve of evidence-based dentistry papers. However, the number of EBD papers did increase in line with the total number of “TMD” papers. In the recent decade, EBD papers also increased substantially to approximately 180 papers yearly. It might be prudently concluded from these data that apart from the 21 papers presented in Table 3, the EBD papers were apparently not proportionally focused on “occlusion”.

A clear discrepancy is visible between the periods of increased activity in the number of papers of the topics “TMD” + “occlusion” and the EBD curve, whereas the curves of “TMD” + EBD and the trial curves of “TMD” + “occlusion” are fluctuating more or less constantly over the 40-year period (EBD papers ranging from 0 to 8 with a top in 2010 and “occlusion” trials ranging from 0 to 7 with a top in 2003, resp.).

The increase of the number of EBD studies is not followed by a progression of both the studies of “TMD” + occlusion (all trials) (*n*=86) and the “TMD”-related EBD (*n*=58). Regarding the 11-year period of increased attention to the “occlusion” and “TMD” topics from 1982 to 1992, all 14 exclusively “occlusion”-oriented RCTs were published. This is the same number of RCTs as that in the 22-year period from 1993 to 2015.

Figure 2 clearly shows the similarity of the curves of evidence-based medicine (EBM), evidence-based dentistry (EBD), and the topic “pyramid of evidence” (PoE), popular terms in today's research (the correlation between the number of publications over the years is, resp., EBM-EBD: 0,922, EBM-PoE: 0.900, and EBD-PoE: 0.934). All 3 topics became more important as the subject of research and scientific interest in the past 2 decades. The onset of interest in evidence-based research papers started in 1995.

In 1977, one single paper with the keyword EBD, two EBM papers, and zero PoE papers were found. In 1995, at the start of the “hype,” 9 EBD papers, 154 EBM papers, and 7 PoE papers were published. In 2015, the number of papers substantially grew to 221 for EBD, 5689 for EBM, and 20 for PoE yearly.

Finally, in a search focused on the topic “Trials” in the Medline database in the period 1950–2017, more than half a million (clinical/controlled/randomized controlled) trials appeared to be present. Refined with the keywords “Evidence Based Dentistry,” 417 studies could be selected. A further refining with “occlusion” resulted in 8 studies.

A summary of all trials in the Medline database in the period 1950–2017 refined with the search terms Evidence Based Dentistry and Occlusion:

1. “Evidence-based clinical practice guideline for the use of pit-and-fissure sealants” by Wright et al. [80]
2. “Does altering the occlusal vertical dimension produce temporomandibular disorders? A literature review” by Moreno and Okeson [69]
3. “Is there enough evidence to regularly apply bone screws for intermaxillary fixation in mandibular fractures?” by Bins et al. [81]
4. “Occlusion on oral implants: current clinical guidelines” by Koyano and Esaki [82]
5. “Bilateral balanced articulation: science or dogma?” by Farias-Neto and Carreiro [83]
6. “Complete denture occlusion: an evidence-based approach” by Farias-Neto and Carreiro [84]
7. “Critical appraisal of methods used in randomized controlled trials of treatments for temporomandibular disorders” by Fricton et al. [65]
8. “Association between orthopedic and dental findings: what level of evidence is available?” by Hanke et al. [85]

As a result of this search, only 2 papers addressing the “occlusion” topic of our study were found: the Moreno/Okeson study and the Fricton study. After replacing “occlusion” by “TMD” in the second refining step of the same EBD search, this revealed only 3 studies: the Keenan 2015 study [86], the Forssell and Kalso 2004 study [87], and again the Moreno/Okeson 2015 study [69]. In the perspective of our study, the study of Forssell entitled “Application of principles of evidence-based medicine to occlusal treatment for temporomandibular disorders: are there lessons to be learned?” is also relevant.

## 4. Discussion

Despite the substantial number (2419) of published papers about “TMD” and “occlusion,” there are still controversy and contradictory opinions on the interaction between “occlusion” and “temporomandibular disorders.”

What could be an explanation for the still ongoing discussion? Why has the scientific world not yet reached consensus? For a one-to-one link between a disorder and a factor, the 2419 occlusion papers, or otherwise, the 52 RCTs, the highest level of research in the pyramid of evidence [88], should have been more than sufficient to elucidate the link between both. However, research is still going on, and the number of papers is still increasing.

A possible explanation for the ongoing controversy about “occlusion” and “temporomandibular disorders” is definition-based.

Ever since Goodfriend described functional disturbances of the stomatognathic system in the Dental Cosmos of 1932 [89], several terms were used to describe deviations from the optimal and healthy normal status of the stomatognathic system. Currently, the term “temporomandibular disorders” (TMDs) is generally accepted and most frequently used to represent disturbances and dysfunction of the stomatognathic system (Table 1). Moreover, in 2014, this term became the “golden standard” for the disorder's diagnostic criteria and taxonomy through a series of workshops and symposia, a panel of clinical and basic science pain experts, reaching a consensus to differentiate TMD into 5 pain-related temporomandibular disorders (3 disorders of muscular origin, 1 of joint origin, and 1 TMJ headache-provocated disorder) and 5 intra-articular temporomandibular disorders [90].

Considering the topic “occlusion” in the dental literature, this term is used for 4 different entities: (1) the anatomic or “orthodontic” jaw relation: the Angle classification, (2) static contact between the teeth of the upper and lower jaws, (3) dynamic contact between the teeth of the upper and lower jaws, for example, cuspid guidance versus group function, articulation, and occlusal interferences, and (4) the prosthetic classifications, more specifically, the complete/incomplete dentition versus complete dentitions and the presence of fixed/removable prosthetics.

Based on purely statistical fundamentals, there are at least 10^4^ = 10,000 different possibilities for research and RCT studies (TMD: the 10 distinguished disorders as described by Schiffman et al. in combination with the 4 occlusion entities).

It can be concluded that due to the phenomenon of the multiple catch-all or container concepts of both “occlusion” and “TMD,” there are many different options to research. In addition, considering the etiology, the cause and effect relation, and vice versa, there are almost inexhaustible possibilities.

In summary, the 52 RCTs of the Medline database over the period 1977–2017 only represent approximately 0.5% of these 10,000 possible study options. In addition, there are also RCTs in this 52-RCT search collection dealing with other topics than exclusively “occlusion.” This is a consequence of the generally accepted multifactorial and multicausal character of “TMD.”

Considering the multifactorial character, 40 years ago, in 1979, De Boever [91] described the well-known multifactorial etiological approach for CMD. He distinguished five theories: the mechanical displacement theory, the neuromuscular theory, the psychophysiologic theory, the muscle theory, and the psychological theory. De Boever stated that none of the theories as such give an adequate explanation of the cause and the symptoms of CMD. He concluded that the etiology of functional disturbances is multifactorial and is a combination of dental, psychological, and muscular factors.

This is in line with the “biopsychosocial” theory published in 1987 by Marbach and Lipton, “Biopsychosocial factors of the temporomandibular pain dysfunction syndrome. Relevance to restorative dentistry” [92]. More recently, Ohrbach and Dworkin published a paper with the modern multifactorial approach, presenting the biopsychosocial model of illness, addressing more focus on the psychosocial domain [76].

**Figure 3:**
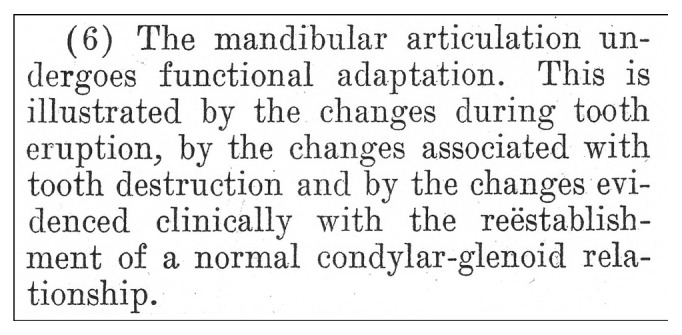
Original fragment in *Dental Cosmos*, volume LXXIV, no. 6, June page 534, 1932.

Considering the adaptation capacity, already in the early years of dental literature in 1932, 85 years ago, Goodfriend stated the following in his concluding remarks: *“The mandibular articulation undergoes functional adaptation”* [89] (Figure 3).

In 2005, Michelotti et al. accordingly wrote the following: *“None of the subjects developed signs and/or symptoms of TMD throughout the whole study, and most of them adapted fairly well to the occlusal disturbance”* [35]. Recently, in 2015, with respect to the capacity of the stomatognathic system to adapt to a recovered or new vertical dimension, Moreno and Okeson wrote the following: *“Permanent occlusal changes should only be attempted after the patient has demonstrated adaptability at the new vertical dimension”* [69].

The stomatognathic system of patients appears to be able to accept and adapt to occlusal alterations.

In conclusion, for almost a century, the scientific world confirms and agrees with the existence of the adaptation capacity of the structures of the stomatognathic system.

Focusing on the aim and the subject of this paper—research about the contradictory role of “occlusion” in relation to “TMD”—the following conclusions about “occlusion” in relation to “TMD” can be abstracted after a more in-depth study of the 52 selected RCTs and the results of the other described searches:

- The role of occlusion in the etiology of “TMD” is not absolutely assessed.
- Occlusal interferences affect “TMD.”
- “TMD” is multifactorial, and subsequently, it will be affected by different treatment modalities (the biopsychosocial model of illness approach).
- “TMD” fluctuates over time.
- Adaptation is an important quality of the human being; in this respect, it is more specific of the stomatognathic system.

The still existing confusion and contradiction in the dental literature about the role of “occlusion” in “TMD” is probably caused by the approach of some mainly American gnathologists/researchers who maintain that since occlusal interferences affect TMD, all occlusal varieties and “abnormalities” cause TMD signs or symptoms and therefore have to be treated preventively. Such an approach to patients does not account for the interindividual variation, as present all over the world and in this context as described by Ramfjord and Ash [93, 94].

Since it will take about a generation to fundamentally change treatment strategies and opinions [95], the profession will still be confronted in the next decades by the often-used concluding statements, sentences, and words in scientific papers: “more research is still necessary,” “the contradictory role of occlusion,” and “the controversy.”

With respect to the possible role of “occlusion” in the etiology of “TMD,” the scientific world has to accept that it will most probably never ever be more elucidated than it is at present. In all probability, there will never be an ethics commission approving “occlusal experiments” in healthy young people to study the onset or incidence of “TMD” signs or symptoms over time.

The only possibility to make any progress is to study in detail the available studies and to respect and implement practitioners' experiences.

It is one of the challenging and obliging tasks of universities, researchers, and dental societies to achieve this progress by performing an objective, accurate, and critical study of the existing literature. This will then result in studies with a sound methodological standard. Accurate reviewing of submitted papers in peer-reviewed journals is also important to achieve this aim. All this might result in less “scientific” papers and consequently less (governmental) granting, but on the other hand, also in a higher level, standard and quality of the published papers.

It will create an achievable way for practitioners to stay up-to-date with the literature to the application of evidence-based dentistry in their daily dental practice and not be overwhelmed by an ocean of conflicting information and endless discussions about scientific topics.

The conclusion of this review is to stop trying to find the exact etiologic role of “occlusion” in the perspective of “TMD” and concentrate on a critical study of the available scientific and clinical information and integrate them.

## 5. Management Section

Based on the available “TMD” papers and experiences from the daily dental practice, first, some general elementary starting issues are detailed below to provide the general practitioner some support and clues for the treatment of TMD patients. Subsequently, practical clinical examples and tips of each issue are presented:

1. Each patient is unique.
2. Respect the biological variation in form/appearance and (the coherent) function.
3. Adaptation is possible within defined biologic limits and contains a time factor or aspect.
4. Be alert to recent (dental) treatments or events that might interrelate with the onset of the complaints or problems.
5. Try to differentiate and diagnose the different entities of TMD and adjust your treatment modality accordingly.
6. Be reluctant with irreversible treatment options and direct the treatment as much as possible to a predictable, reliable, and proven result with a known determined prognosis.
7. Consider and take into account the opinion or idea of the patient about the possible cause of the complaint or problem.

Observe and consider that not only the anatomy and morphology of each individual diverge but also the regular everyday use of the stomatognathic system differs from one another. The biologic variation is huge, even within distinguished ethnic groups. The functions of the stomatognathic system: communicating (talking, laughing, kissing, and making love), eating (biting and chewing), supporting (TMJ's orthodontic abnormalities), stress processing (bruxism, grinding, and clenching), and aesthetics, differ significantly between individuals. There may be clues present between the complaints and the observed problems with the functions of the stomatognathic system of the patient in question. The patient's age and gender also affect the prevalence of TMD (De Kanter et al. [96] and, more recently, Lovgren et al. [97]). Also, women are on average smaller than men, have less muscle mass, and have on average a more limited maximum mouth opening. In daily life, they will meet the limits of function and maximum mouth opening more frequently than men. For example, US Big Macs and French baguettes have the same size for both genders. Also, some physical intimacies and sexual activities in the most prevalent heterogeneous relationships for men and women differ substantially with respect to the maximum mouth opening and the limits of the temporomandibular joints [98].

Adaptation is possible within certain biological limits; however, including the accessory time component hereby is an important and essential factor. Clinical experience reveals that this time component influences the eventual exceedance of adaptation in two ways. In patients with restored dentitions, the presence of different restoration materials, materials with a different hardness and wear component, will not wear equally over time. As a result, even after a long period of the application of the restoration, the tooth restored with the most resistant and hard restoration material might cause an uncomfortable feeling, become more or less painful, and provoke complaints and TMD resembling signs or symptoms. These strong tooth-related complaints are difficult to distinguish from endodontic problems. Apical X-rays might be an aid to reveal the correct diagnosis in these cases. For one reason or another, no (further) intrusion of the concerning tooth occurs as a possible mechanism of compensation or adaptation. This tooth also apparently does not abide the overall biological, natural, and functional wear of the other adjacent teeth. Subsequently, as a mechanism of adaptation, their mobility increases slightly. These patients indicate most of the time that the tooth is feeling a little bit higher and more sensitive. Based on clinical experience, these, a little more than physiologic mobile teeth, show most often interferences at chewing movements, at articulation, and not in the static occlusal contact position. These are mostly indirect restorations such as (solitaire/single) crowns, abutment teeth for removable casted partial prosthetics, or the occlusal clasps of removable partial dentures. These so-called “iatrogenic occlusal interferences” will manifest only over time. It is advisable to eliminate the disturbance by selective grinding and reshaping and adjusting the contour of these restored teeth or clasps. The recently developed device “Tekscan®” may be a useful aid to substantiate this, also in the treatment of “occlusion-sensitive” subjects [99].

Exceeding the adaptation capacity might also occur in a specific and limited period of time, during the eruption of the wisdom molars, more specifically, the eruption of the 3rd mandibular molars. Although the eruption pattern of the 3rd molars shows a wide range, the majority erupts between the age of 17 and 26 years [100, 101]. Especially when the process of the eruption is slightly disturbed, occasionally, the distal part of the second molar might become dislocated and pressed up distally resulting in a slight tipping along the mesiodistal axis and cause a physiologic-based occlusal interference in the most posterior region of the mouth. The closer a (slight) static occlusal interference is to the temporomandibular joints, the bigger will be the change in the vertical dimension and influence on the closure of the mouth especially in the anterior region. As a consequence, this will affect the musculature. Furthermore, the TMJs will go out of balance as a compensation mechanism, deflecting from their physiologic position. Any outmost slight change in the vertical dimension posterior will have more impact on the TMJs and the musculature and result in a more extreme change in the vertical dimension than would ever be possible with the same dimensional changes more anterior.

The distal interference causes tilting of the concerning TMJ and might initiate grinding and wear of the contralateral cuspids in a short period of time, as a parafunction, mostly unconsciously at night. In combination with the mostly irritated operculum, patients compensate for this with an unnatural, divergent chewing pattern. This unnatural chewing pattern occurs in unnatural positions within the TMJs and the articular discs, resulting in dislocation, joint sounds, and pain in and around the TMJs. Counseling, information, advice about extraction, or preferably the extraction procedure itself is desirable. Some adaptation time after the extraction of the third molar, the affected and worn top of the cuspid might be restored using the composite etch technique. If, for any reason, the third molar will not be extracted, treatment of the painful operculum is recommended. Both the further eruption of the third molar and the occlusal condition have to be monitored or treated.

With respect to the onset of TMD or TMD-related complaints, it is advised to be alert to recent dental procedures or events that might be associated with the complaints. This includes extractions, particularly extractions of mandibular teeth. Damaging or even luxation of the TMJs might have occurred. Also, long-lasting dental treatment procedures when the patient had to have the mouth opened extremely wide and for a long time may have this effect. Equally, the intubation procedures for general anesthesia/surgical interventions are notorious and suspected. As a result, the TMJs might have been extremely strained and stretched, and the musculature might have been traumatized and injured. Recovery of these attacks on the TMJ tissues requires time and has to be treated by getting as much rest as possible. This means no excessive function, temporary use of soft or liquid foods, and/or temporary medication for pain relief.

Recently inserted prosthetic devices also might provoke complaints of a TMD character such as tenderness of the chewing muscles, biting on the cheek or lips during chewing, joint sounds, or even pain in or around the TMJs and the masticatory muscles. In case, after accurate inspection, no shortcomings or imperfections of the prosthetic provisions and no deviations in the static and active dynamic occlusion can be determined, the adaptation capacity has to be appealed. The patient has to be informed and counseled and explained that more time is necessary to adapt to the new situation. Not infrequently, the patient is not convinced of this approach and appears to be unhappy with the aesthetics or comfort of the new devices. The patient explains and interprets this by a lack of the support function and the presence of chewing problems.

Frequently, the patient's interpretation of those shortcomings is gratefully strengthened by the treatment and repetitive corrections of the occlusion by the general practitioner. A treatment not addressing the real cause or problem will never ever be successful. In extreme cases, placebo adjustments might be applied to address and permeate this problem.

From a historical perspective, TMD patients show different, nowadays better distinguished functional disorders. It is important for the dental practitioner to assess the most possible specific diagnosis. If the diagnosis is correct, a conforming, matching therapy and treatment is available. If the treatment was successful, then the diagnosis was the right one. Crucial for this is a good and complete examination of the patient. Anamnesis and clinical examinations, not only intraoral but also from the head and neck region, are very important and indispensable.

Important signs might be observed as nonverbal expressions such as the overall body posture, the position of the head in relation to the chest, shoulders up or down, and patients' handshake with a firm or soft hand. How does the patient communicate? How clearly does he or she express himself or herself? Is there a (recent) trauma in his or her history? How many different professionals have previously been consulted? Is the location of the pain clearly pointed to with one finger, for instance, the joint, or do the patient's hands encircle the entire head? Are other joint problems present such as hypermobility of knees, ankles, wrists, or elbows? Is there a history of rheumatic diseases?

All this information is important to assess a correct and specific diagnosis.

Whenever the general practitioner is not able to reach a right circumscribed diagnosis, other experts have to be consulted before any treatment is proposed. The advice of a specialized gnathologist might be required, or a physiotherapist specialized in TMJ problems might be consulted mainly for muscular problems, or a (clinical) psychologist.

There is plenty of literature available describing the patient's anamnesis and clinical examination protocols [102, 103].

Occasionally, even after intensive examinations and interdisciplinary consultation, a general practitioner might not be convinced of a right treatment approach of the specific TMD. In that situation, it is advised to be cautious with (irreversible) treatment options. One has to treat as predictably as possible. In progressive bruxism cases, preventive occlusal splints are necessary. The application of occlusal appliances is also recommended as indispensable reversible tools to test, restore, and establish a physiologically accepted natural and healthy comfortable balance in the stomatognathic system.

Whenever a patient is not aware of a TMD problem, but the dental practitioner recognizes signs of TMD at the regular checkups of the patient, it is important to find out whether the patient himself or herself has any idea or assumption about the cause or the existence of the assessed TMD phenomenon [104]. If not, it is advised to be very cautious with active treatment, and informing the patient absolutely has to be the first step. Try to formulate understandable and acceptable arguments about your concern as a dental practitioner concerning the patient's dental health and the possible benefit of the proposed treatment. When the patient lacks the conviction about an intervention, it is better to (temporarily) abandon the proposed treatment. In these cases, it is preferred to evaluate and monitor the determined deviation by means of repetitive produced cast models of the dentition or chronological assessed digital files of it.

## 6. Epilogue

This study at the end of the professional career of the first author is the final spin-off from the PhD thesis: Prevalence and Etiology of Craniomandibular Dysfunction. An Epidemiological Study of the Dutch Adult Population [105]. In this present paper, we tried to present research and experiences from the dental practice in a symbiosis. Some final interesting and, in our opinion, wise quotes from experts in this field to remember are the following:

*“More emphasis should be placed on patient-centred criteria of what is perceived to be important to patients' function, satisfaction, and needs, as well as dentists' views of what is significant for improvement in dental health”* Ash [55].

A final comment of Klineberg to the Forssell Paper [106] about evidence-based medicine with respect to TMD occlusal treatment is the following: “*It is clear, that even without a role in TMD etiology, the occlusion retains an important role in most aspects of dental practice”* [106].

And last, but certainly not least, also the most recent statement of these 3 from Carlsson is the following [59]: *“In a longer perspective, many of today's “truths” will be questioned, and dogmas that lack strong evidence will eventually be abandoned. But to achieve this goal it is necessary for open-minded educators and researchers to question and analyse current practice methods in all areas of clinical dentistry.”*

## Figures and Tables

**Table 1:** Web of Science search in the Medline database showing the number of papers of functional disturbances of the stomatognathic system: “TMD” over the period 1950–2017, which is focused on the biopsychosocial model (BPSM) and “occlusion” specified for different types of trials: clinical trial (CT), controlled clinical trial (CCT), randomized controlled trial (RCT), all trials, and evidence-based dentistry (EBD).

Term/topic of the search	All papers	BPSM	Occlusion					
		Papers	Papers	CT	CCT	RCT	All trials	EBD
Craniomandibular disorders	826	2	174	13	3	11	15	2
Craniomandibular dysfunction	448	1	113	8	2	6	8	1
Temporomandibular disorders	14316	31	1533	47	11	37	63	20
Temporomandibular dysfunction	6686	11	1256	40	9	26	45	5
CMD	1848	1	50	3	1	2	3	0
TMD	4802	22	399	13	3	18	25	5

All 6 search terms: “TMD”	21686	35^∗^	2419	69	16	52^∗^	86	21^∗^

^∗^Studies that detailed the subject of this review.

**Table 2:** Web of science search in the Medline database for RCTs in the period 1977–2016 with the keyword “TMD” + “Occlusion” by the first author, characteristics of the trial number of citations, and abstracted summary of the result or effect of the trial.

Author	Year	Characteristics of the trial	Citation	Effect/result	Reference number
Leal de Godoy et al.	2015	Laser therapy–TMD diagnostic criteria	0	No	[26]
Costa et al.	2015	Occlusal appliance–headache	0	No	[6]
Cioffi et al.	2015^∗^	Occlusal interference–EMG muscular activity	0	“Little”	[4]
Rampello et al.	2013^∗^	Universal occlusal appliance–TMD diagnostic criteria	2	“Favorable”	[40]
Yu et al.	2013	Full denture lingualized occlusion–“TMD”	0	“Remission”	[53]
Jakhar et al.	2013	Surgical procedure TMJ–CT evaluation	3	“Improvement”	[18]
Michelotti et al.	2012^∗^	Education/occlusal appliance–musc. pain and mouth op.	32	“Education slightly better”	[36]
Ap Biasotto-Gonzalez et al.	2010	Food texture–EMG activity	1	“Less variation”	[54]
Ueda et al.	2009	Jaw excercises–OSA	11	“Help relieve”	[48]
Hamata et al.	2009^∗^	2 types of occlusal splints–TMD clinical and EMG	10	“Remarkable reduction”	[17]
Diernberger et al.	2008	Prefered chewing side–epidemiologic study	37	Several “associations”	[12]
Monaco et al.	2008	Osteopathic manipulative treatment–kinesiographics	12	“Induce changes”	[37]
Toro et al.	2007	Surgical procedures analgesics–jaw movements	6	“A valid aid”	[44]
Conti et al.	2006	2 types of occlusal appliances–TMJ pain	34	“No differences”	[5]
Le Bell et al.	2006^∗^	Occlusal interferences–subjective sign of TMD	29	“Stronger symptoms”	[25]
Wolfart et al.	2005	Prosthetic appliance–SDA and molar occlusion	37	No	[52]
Ueki et al.	2005	Surgical procedures–skeletal stability	32	No/“similar”	[49]
Michelotti et al.	2005^∗^	Occlusal interference–TMD signs and symptoms	47	No/“adapted fairly well”	[35]
Magnusson et al.	2004^∗^	2 types of occlusal appliances–TMD signs and symptoms	28	“Some or significant”	[31]
Fayed et al.	2004^∗^	2 types of occlusal appliances–magnetic resonance	11	“Effective, one superior”	[13]
Turp and Schindler	2003	Refer to the trial of Le Bell 2002–descriptive study	7	“No new data”	[47]
Le Bell et al.	2002^∗^	Occlusal interferences–TMD signs and symptoms	40	“Significant”	[24]
Maloney et al.	2002	Jaw movement devices–TMD signs and symptoms	22	“Effective”	[32]
Bettega et al.	2002	Surgical procedures–correct occlusion	23	“Differences”	[3]
Raphael and Marbach	2001	Occlusal appliances–widespread body pain	69	“Improvement”	[41]
Glaros et al.	2000	Biofeedback–TMD pain	32	“Significantly higher”	[15]
de Andrade et al.	1998	Surgical procedures–TMD signs and symptoms	13	Significant	[11]
Kirveskari et al.	1998^∗^	Occlusal interferences–TMD signs and symptoms	41	Significant	[21]
Rodrigues-Garcia et al.	1998	Orthodontic surgical procedures–TMD signs and symptoms	45	“Other factors responsible”	[43]
Davies and Gray	1997^∗^	Occlusal appliances' wearing time–TMD signs and symptoms	19	“All marked improvement”	[10]
Karjalainen et al.	1997^∗^	Occlusal adjustment–TMD signs and symptoms	14	Significant	[20]
Obrez and Stohler	1996	Muscle irritation–range of mandibular movements	36	Significant	[38]
Vallon et al.	1995^∗^	Occlusal adjustment–TMD signs and symptoms	16	Significant	[50]
Tsolka and Preiskel	1993^∗^	Occlusal interferences–EMG and kinesiographics	20	Not significant	[46]
List and Helkimo	1992^∗^	Acupunture/occlusal appliance–TMD signs and symptoms	42	“No differences”	[28]
Tsolka et al.	1992^∗^	Occlusal adjustment–TMD signs and symptoms	21	“No differences”	[45]
Lundh et al.	1992^∗^	Occlusal appliances–TMD signs and symptoms	77	“No differences”	[29]
Johansson et al.	1991^∗^	Acupunture/occlusal appliance–TMD signs and symptoms	65	“No differences”	[19]
Gray et al.	1991^∗^	Occlusal appliances–TMD signs and symptoms	12	“No differences”	[16]
Kirveskari et al.	1989^∗^	Occlusal adjustment–TMD signs and symptoms	29	Yes/no significant	[22]
Lundh et al.	1988	Occlusal therapy/appliance–disk displ. with reduction	55	“Differences”	[30]
Lipp et al.	1988	Intubation procedures–TMD signs and symptoms	7	“Temporary effect”	[27]
Wenneberg et al.	1988^∗^	Occlusal adjustment/appliance–TMD signs and symptoms	34	“More effective”	[51]
Puhakka and Kirveskari	1988^∗^	Occlusal adjustment–globus symptoms	20	“Significant association”	[39]
Forssell et al.	1986^∗^	Occlusal adjustment/appliance–TMD signs and symptoms	30	“Effective treatment”	[14]
Raustia	1986	Acupuncture/tomography stomatognathic treatment	6	Paper not available	[42]
Kirveskari and Puhakka	1985^∗^	Occlusal adjustment–globus symptoms	11	“Significant association”	[23]
Manns et al.	1985	Occlusal apliances–EMG activity	30	“Study suggests”	[33]
Dahlstrom and Carlsson	1984^∗^	Biofeedback/occlusal apliance–TMD signs and symptoms	25	“No differences”	[9]
Dahlstrom	1984^∗^	Biofeedback/occlusal apliance–TMD signs and symptoms	3	“A positive correlation”	[7]
Manns et al.	1983^∗^	Occlusal appliances–EMG activity	52	“More effective”	[34]
Dahlstrom et al.	1982^∗^	Occlusal appliance/biofeedback–TMD signs and symptoms	36	No significant differences	[8]

^∗^Exclusively occlusion-orientated studies.

**Table 3:** Web of Science search in the Medline database in the period 1950–2017 for all “TMD” terms (*n*=21,686) refined with the keywords “Evidence Based Dentistry (EBD)” (*n*=60) and “Occlusion” (*n*=21) chronologically by the first author, title/characteristics of the study, number of citations, type of the study, and reference number [55–75], which is presented in a chronological order of the year of publication.

First author	Year	Title/characteristics of the study	Citation	Study type	Reference number
Weinberg	1976	TMJ function and occlusion concepts	23	Article	[74]
Becker	1995	Occlusion etiology of TMD	5	Article	[56]
Ey-Chmielewska	1998	Ultrasonic techniques for painful TMD, with ultrasonic exam aid	1	Comp. + CT + CCT	[64]
Ash	2001	Paradigms of TMD and occlusion	18	Review	[55]
Gremillion	2002	Orofacial pain, a pain-oriented study	6	Review	[66]
Rinchuse et al.	2005	EBD versus experience-based views on TMD + occlusion	26	Article	[72]
Dawson	2005	“EBD-based versus experience-based views on occlusion and TMD”	2	Letter + comment	[60]
Rinchuse and Kandasamy	2006	Centric relation: orthodontics	35	Review	[71]
Luther	2007	TMD and occlusion: orthodontics	11	Review + evaluation	[68]
Carlsson	2009	Review of prosthodontic dogmas	72	Review + meta-analysis	[58]
Carlsson	2010	TMD and occlusion dogmas	27	Review	[59]
Fricton	2010	Critical appraisal of TMD-RCTs	14	Meta-analysis	[65]
Blackwood	2010	“After 50 years in practice, the evidence is convincing”	0	Letter + comment	[57]
Roehm	2010	“Gnathology lessons from a 1969 Oldsmobile engine”	0	Letter + comment	[73]
Hudson	2010	“Myths of orthodontic gnathology”	0	Letter + comment	[67]
Pensak	2011	“One has to wonder”: orthodontic and neuromuscular balance	0	Letter + comment	[70]
Donovan	2014	Annual review of the American Academy of Restorative Dentistry	1	Review	[62]
Wiens and Priebe	2014	Occlusion article: occlusal concepts in prosthetic dentistry	5	Review	[75]
Moreno-Hay	2015	Occlusal dimensions: a review	4	Review	[69]
Donovan et al.	2016	Annual review of the American Academy of Restorative Dentistry	1	Review	[63]
de Kanter	2016	TMD prevalence and etiology: a historical article in Dutch	0	Review	[61]
